# Circular Medicine – Being Mindful of Resources and Waste Recycling in Healthcare Systems

**DOI:** 10.2147/RMHP.S396667

**Published:** 2023-02-21

**Authors:** Richard Syms, Simon D Taylor-Robinson, Guglielmo Trovato

**Affiliations:** 1 Department of Electrical and Electronic Engineering, Imperial College London, London, UK; 2 Department of Department of Surgery and Cancer at St Mary’s Hospital Campus, Imperial College London, London, UK; 3 Faculty of Medicine, University of Catania, Catania, Sicily, Italy

**Keywords:** circular economy, recycling, climate change

## Abstract

In the light of the COP27 Climate Change Conference, the concept of the circular economy has come to the fore with promotion of reuse and recycling of appliances and materials from electronics to clothes. This concept has not been widely taken up by healthcare systems. In this perspective article, we discuss the idea of the circular economy and how, by extension, the concept of “circular medicine” with optimised hospital and medical clinic waste recycling might be promoted in the context of better stewardship of resources in healthcare management.

## Introduction

The concept of the circular economy is gaining popularity with an attempt by manufacturers and consumers to at least pay lip service to recycling and re-use of materials in order to reduce the burden of waste products piled high in refuse mountains all around the planet.^1^,^2^ The strategy seems to share attributes with current movements centred on climate change and pollution, namely, to be socially worthwhile and to have a strong pressure group, defined goals and terminology, but it currently appears to offer few practical solutions.^1^,^2^

From this broad idea of being more careful with resources and how they are managed, the “circular economy” can be extended to the healthcare arena with the concept of “circular medicine”. This is particularly important as hospitals are large producers of waste or unwanted products and very little is reused or recycled in a sustainable way.^3^

## The Current Situation in the Wider Economy

The unfortunate truth is that little of any product currently gets re-used, re-cycled, or re-purposed in the affluent West. Individual behavioural patterns are more influenced by desires and cost than by ethics or any need to balance future consequence against short-term gratification.^3^ These patterns are reflected in industry. One egregious example is fast fashion, where the fashion industry has deliberately encouraged single use of products with an intrinsically long lifetime, in order to maximise sales.^4^ Another is the information technology (IT) industry, where continual software “upgrades” force hardware upgrades long before end-of-life. Legislation (for example, the waste electrical and electronic equipment (WEEE) and restriction of hazardous substances (RoHS) directives for electronics) and tax incentives (recycling R&D tax credits) are often the only ways to prevent some behaviours and encourage others, but they often achieve little against industry lobbies.^5^

There is in addition a culture of self-deceit regarding recycling.^6^ For much domestic and industrial waste, recycling often involves collection, sorting and separation of items that are subsequently simply dumped from the Global North on the Global South.^1^ Only items predominantly comprising a single bulk material can be recycled cheaply; disassembly is generally uneconomic.^6^ Obvious examples are wood, paper, clothing, bottles, cars, aircraft, and ships, from which wood pulp, textiles, glass, and metals such as aluminium and steel can easily be recovered. All have been recycled for many decades.

In some cases, simple automatic separation techniques exist, for example based on colour (for glass) magnetic properties (iron and steel) or eddy currents (aluminium, copper and other non-ferrous metals). Other processes are much more costly. Disassembly generally requires manual methods that often (especially for ships) also require initial transport to a low-wage economy. The most pernicious “new” materials - plastics - can only be recycled as degraded polymers, even after sorting by polymer type and colour.^6^ Other large-volume materials (for example, reinforced concrete building materials) cannot be transported long distances or recycled at all.

Electronics can be and are re-cycled, but due to the wide variety of circuitry, very high levels of integration and the need for specialist knowledge the process is expensive except for semi-standard products such as PCs.^5^ Components must be separated from printed circuit boards (PCBs), sorted, and tested, and, even then, may fail to satisfy reliability criteria.^5^ However, recovery of high-value components (processor chips, memory chips and image sensors) may still be worthwhile.

These aspects have assumed much greater significance in the developed world during the COVID-19 pandemic, due to failures of the globalised supply chain. The importance of electronics has remained high because of the continued failure of China to successfully exit the pandemic. They may assume even greater significance if Taiwan (the main source of many high-value electronic components) has escalating conflict with China, or if its semiconductor fabrication plants (“fabs”) are demolished prior to a putative Chinese take-over (the “broken nest” strategy). The combined result will likely be a worldwide re-appraisal of low-cost production of strategic items in distant countries.^3^,^6^

Some items can easily be re-purposed. The most obvious examples are buildings, which are often re-purposed for many centuries. In many other cases, only very high-value items are worth re-purposing. Many of these are military and are continually upgraded. Aircraft and ships enjoy extended careers and can still be repurposed at the end of their military life.

## How Might Any of This Affect Medicine?

The main unique factor is the obvious health risk of potentially contaminated items that do not arise in the more general circular economy.^2^ Widespread litigation (especially in the US) drives risk-averse behaviour, making disposal the most likely outcome. There is in addition legislation to prevent many items reaching unqualified hands, and a deliberate lack of technical support for second-hand equipment. However, some common medical items might be re-cycled, re-used, or re-purposed as follows:

- Hospitals have a history of not being re-purposed; they are almost always re-built to exploit existing land, but they take account of changes in disease patterns (such as the decline of tuberculosis and polio) and the introduction of new medical practices (diagnostic imaging).
- Ambulances can easily be re-purposed, the most obvious recent example being the supply by the West to Ukraine of refurbished field ambulances.
- Bedding and hospital clothing can be re-used almost indefinitely through laundry and sterilization.
- Dressings and PPE are generally use-once but can generate electricity through large-volume incineration.
- Needles, syringes other sharps are universally incinerated, some the most serious medical disasters having arisen from needle and syringe sharing (for example, the tartar emetic injection disaster in Egypt, which led to an epidemic of hepatitis C).^7^ As a result, some countries (again Egypt) have planned to introduce self-destructing syringes to force single use.^8^
- More expensive surgical tools and endoscopes are routinely re-used after specialised cleaning and sterilization, presumably almost indefinitely.
- Lower-cost catheter tools can also be re-used after sterilization, but presumably only until the risk of mechanical failure outweighs the benefits of any cost saving.
- Orthopaedic implants although potentially recyclable are not re-used due to taboos around cadavers. Electronic implants could be re-used, but since many (pacemakers, DBS systems) are safety critical, the risks may likely outweigh the benefits.
- Artificial limbs are patient specific, but could be re-used with simple mechanical modifications and would be of high value in historic war zones.
- Spectacles may easily be re-used after sorting with simple optical instruments; hearing aids could also easily be re-used after mechanical adaptation of mouldings.
- Small electrical equipment such as ultrasonic imagers, monitors for heart rate and level of anaesthesia, and electrosurgery tools fall into a similar category as conventional electronics; the economics of disassembly concentrates re-cycling on to high-value components.
- Large electrical equipment such as X-ray, CT, MRI, and PET scanners often contain expensive parts such as magnets and radiofrequency detectors that could easily be re-used. However, none are particularly portable. There are obvious dangers associated with disassembly of X-ray or CT systems containing radioactive sources. However, re-use of lower-field MRI systems in countries with less developed health systems is both practical and worthwhile.

We would recommend that hospitals, for example, make it policy to engage with suppliers who have sustainability policies both in production, but also in waste management. Partnerships between healthcare suppliers and hospitals aimed at recycling or reuse of waste or unwanted products should be incentivised at individual institutional, local, regional, national and international levels.

## Conclusion

In the wake of the COP27 Climate Change Conference in Egypt, healthcare systems should be more mindful of the circular economy and the need to minimise the waste produced.^9^ Management strategies and training of staff to accomplish these goals should be instituted with the help of manufacturers and suppliers. We would suggest that hospitals are encouraged only to engage with suppliers who have adopted true sustainability policies.
